# American Dental Association

**Published:** 1891-03

**Authors:** 


### Reports of Society Meetings.

### AMERICAN DENTAL ASSOCIATION.—THIRTIETH ANNUAL MEETING, EXCELSIOR SPRINGS, MO., AUGUST 5 TO 8, 1890.

(Continued from page 117.)

*Thursday Evening's Session.*

The session of the American Dental Association, Thursday evening, was held at the Opera-House at Excelsior Springs, Mo. The first business was the election of officers, with the following result: President, Dr. A. W. Harlan, of Chicago; Vice-President, Dr. J. D. Patterson, of Kansas City, Mo.; Second Vice-President, Dr. H. B. Noble, of Washington, D. C.; Recording Secretary, Dr. George H. Cushing, of Chicago, Ill.; Corresponding Secretary, Dr. Fred. A. Levy, of Orange, N. J.; Treasurer, Dr. A. H. Fuller, of St. Louis, Mo.

Dr. W. W. Allport, of Chicago, moved that the question of the place of the next meeting be left with the Executive Committee. This elicited considerable discussion, and the appointment of a board of business managers was suggested.

Dr. A. W. Harlan was then escorted to the chair. He briefly thanked the Association for the honor conferred upon him and assured the members that he would work for the best interests of the organization.

A resolution complimenting the management of the hotel and Opera-House at Excelsior Springs for the favors to the Association was next introduced and unanimously carried.

Dr. Hunt, of Iowa City, Iowa, then addressed the convention upon the blood-supply of the teeth. Dr. Hunt explained that the accepted idea concerning the blood-supply of the teeth is that it is derived from the maxillary arteries by branches which pass directly from the artery to and through the foramina of the various teeth. Dr. Hunt's position is that the supply is not direct. He holds that it passes from the artery through the bone of the maxillary processes by circuitous routes to the pericementum, from which it is distributed by what may be called a capillary system to the foramen. He claims to have demonstrated positively this idea in the forming tooth, and sees no reason to believe that there is any change in the supply to the formed tooth. Dr. Hunt claims also that the human tooth has often more than one foramen. The lecture was illustrated by a magic lantern and was full of original ideas and new and novel points.

Dr. W. X. Sudduth then closed the evening's session by a lecture, also illustrated by the magic lantern, devoted to tumors of the mouth.

At the final meeting of the National Association of Dental Faculties the following officers were chosen: President, Dr. L. D. Carpenter, Atlanta, Ga.; Vice-President, Dr. W. H. Evans, St. Louis, Mo.; Secretary, Dr. J. D. Patterson, Kansas City, Mo.; Treasurer, Dr. A. H. Smith, Cincinnati, Ohio.

*Fourth Day's Proceedings.*

The fourth day's proceedings of the American Dental Association were held at the Excelsior Springs, Mo., Opera-House, Friday, August 8, the newly elected president, Dr. A. W. Harlan, of Chicago, Ill., in the chair.

The committee appointed to arrange for the Dental Congress at Chicago in 1893 elected as permanent officers, Dr. W. W. Walker, of New York, president; Dr. A. O. Hunt, of Iowa City, Iowa, secretary; and Dr. Marshall, of Chicago, Ill., treasurer.

A resolution of thanks to President Foster and the retiring officers was unanimously adopted.

Dr. C. N. Peirce, of Philadelphia, moved a reconsideration of the vote upon the communication of the S. S. White Dental Manufacturing Company. Dr. J. N. Crouse, of Chicago, Ill., explained that the reconsideration was intended for the purpose of striking out all of the report except the conclusion arrived at by the committee in the summing up. The objection was raised that only a few members of the Association were present, and that a reconsideration at this time would probably call forth considerable criticism from those not present. The motion was lost.

A resolution was offered instructing the Executive Committee to send out circulars to the local societies for the purpose of increasing the interest in the work of the American Dental Protective Association. The resolution was adopted.

A resolution was read by Dr. Patterson, of Kansas City, Mo., to the effect that the committee of three appointed to investigate the merits of the recent decisions of the New Hampshire Supreme Court affecting dental boards be instructed to see that the case is properly defended in the courts, and authorized to draw on the treasury to the extent of $500.

Dr. Crouse, of Chicago, Ill., on behalf of the American Dental Protective Association, wanted the president authorized to appoint members of the Association to the vacancies whenever they occur in the committee having this matter in charge. A motion to this effect was carried without opposition.

Dr. L. Jack, of Philadelphia, and Dr. J. Y. Crawford, of Nashville, Tenn., presented a resolution to the effect that a board of business managers be selected to fix the place of the next meeting and perfect arrangements of a general nature. This resolution was decided to be out of order, and that the matter could only be reached by an amendment to the constitution.

Dr. M. L. Rhein, of New York, referred briefly to the paper presented to Section 7 by Dr. Hugenschmidt, of Paris, France, after which the section was passed. Section 4 was also passed, and Dr. W. B. Ames, of Chicago, Ill., presented the report for Section 1. This section is devoted to prosthetic dentistry, chemistry, and metallurgy. Dr. Ames is the secretary of the section and he reported two papers, one by Dr. C. S. Case, of Jackson, Mich., and the other by Dr. E. P. Brown, of New York.

Dr. Case, of Jackson, Mich., addressed the convention upon a system for constructing front crowns.

For the incisor and cuspid teeth, the root is cut off and trimmed as for an old-style wood-pivot tooth; a collar is then made in the usual way, except that it is left broad enough to extend to the cutting edge of the crown. The labial and palatal surfaces of this collar are then cut away in such a manner as to leave a cone-shaped projection on the approximal surfaces to the cutting-edge of the teeth. Into this collar a plate of gold is bent and soldered, making a cap over the end of the root. This is then placed in position on the root and a pin adjusted into the root-canal, if desired, the tooth ground to place and backed with thin platinum and a stiffer gold backing, and the whole cemented together with hard wax. It is then removed from the root, invested, and just enough solder used to attach the pin and tooth to the collar. An impression of the lingual surface of a corresponding natural tooth is then obtained in moldine and a die made of fusible alloy, with which a gold face is obtained, which is fitted and soldered to the lingual surface of the crown, producing a very artistic result. If desired, instead of swedging the lingual face, it may be made with solder at the time when the pin and porcelain are soldered to the collar, but this takes more material and does not produce so artistic a finish.

For bicuspids and molars the roots are cut off square with a bevel on the labial side extending beneath the gum margin. The collar is made in the same manner as for an all-gold Richmond crown. The buccal surface is cut out and as much of the piece used as is necessary to extend over the end of the root to the pulp-canal. This piece is burnished down to the bevelled part of the root and soldered to the collar, furnishing a shoulder upon which the porcelain facing is to rest. The facing is backed and fitted to place and a pin for the root-canal, if desired, adjusted and the whole soldered as described above. The top of the collar and facing are ground off, and a cap swedged, in the usual manner for bridge-work, and soldered. This makes a crown that economizes material, time, and labor in the construction of a bicuspid tooth. It also makes it easy to insert pins into the root-canals, thus securing a more stable setting.

Dr. Case, instead of using platinum backing to cause the solder to flow under the porcelain tooth to make a close joint, uses jewellers' white enamel, which he places in the joint and under the backing, and when the soldering is done it fuses and makes an impervious case.

Dr. P. S. Brown, of New York, described a new form of porcelain crown, the benefit derived consisting chiefly in the shape of the pin, which is round or oval instead of square.

Dr. D. R. Stubblefield, of Nashville, Tenn., then addressed the convention on "Amalgamation and Dental Amalgams."

The speaker reviewed the history of amalgamation, the compounding of one or more metals with mercury. The discoverer of amalgams is unknown. Theophrastus, 300 B.C., seems to have been the first to mention mercury. He calls it liquid silver, from which we get quicksilver. Pliny, in the middle of the first century, mentions that all things float in it except gold, and that alone combines with it. From that time we find statements more or less definite, showing that the knowledge of the solvent power of mercury upon other metals was a laboratory tradition. Dr. Stubblefield then called attention to the experiments of amalgamation, the manner and methods of conducting heat, and the electrical conducticity of amalgams.

The different amalgams, as far as known, are crystalline in structure, and it may be that, instead of dissolving in mercury as salt does in water,—*i.e.*, by the breaking down of the molecule,—the mercury disintegrates the alloy to nothing smaller than the crystal unit, so that when the mass is squeezed through chamois leather the crystal units which have collected in masses of sufficient size cannot pass through the pores of the skin, while the smaller masses and the single crystals are readily forced out.

The history of amalgam from the stand-point of the dentist is not so old. Many of those present will doubtless recall its earliest advent. I have been unable to obtain any authentic account of its first discoverer or the one who presented it first to us. Filing silver coins for us, in this way seems to have constituted the first alloy devoted to it, and yet at this early state no idea of preparing special alloys for the purpose seems to have entered into any mind. At first not only was the use of it looked upon with suspicion, but there was actual enmity and outright opposition. Whether the precarious existence was the cause of something else I do not know, but everything in this connection was vague and uncertain. Those who dared to use it felt guilty, and, like Peter, were ashamed and ready to swear that they did not know it, much less use it, when confronted by the accusation. It was a sort of moonshine business throughout, and the great wonder is that it survived such powerful and undiscriminating persecution. For a long time nothing could be known of its qualities, and if there did not exist the law in nature called the survival of the fittest it would be known to-day only as a tradition, only as a folly that died a natural death. But such is not the case. It has asserted itself, so to speak, in the very teeth of its defamers, and holds to-day a more or less conspicuous place in every office in the land.

The speaker then reviewed the relative merits of the different combinations of tin, silver, gold, platinum. copper, and zinc. In concluding, he said, "I merely wish to say that there are cavities in certain mouths that, from the very nature of the disabilities under which the operator must labor, can better be filled with plastics than anything else. My plea for the material is based upon the fact that it has never had a fair chance. It was born under adverse circumstances and under unpropitious skies, and it was at once relegated in effect, if not in fact, to the kitchen and the scullion. It was not used nor recognized by the most expert operators, and when it was tolerated it was merely to begin an unequal fight for existence as the custodian of a tooth 'not worth anything so expensive as gold.' It has been sneered at and scoffed at since its earliest hour, and yet, like another 'ugly duckling,' it has survived disgrace, and I think the day is not far off when the best and the deftest will hail it as the truest and best friend when the need is sorest. Give it a trial. It is being given a show at last, a fair trial almost everywhere and by everybody, and it will yet convince the most sceptical that it has qualities that render it valuable in many places. A year ago in Galveston I was taught that it was excellent for filling the canals of pulpless teeth. Since that time I have used it carefully, and I am glad to say with better success than anything else I ever used as a root-filling. Dr. Miller, of Berlin, in his tabulated statement of the relative germicide power of the various filling materials, puts copper amalgam at the head. If this is so, and if in root-canals we desire aseptic power present, it seems to stand to reason that the practice is good. At any rate, I state it as true that it has been my friend for the past year in such cases."

Dr. J. Taft, of Cincinnati, Ohio, chairman of the Committee on Necrology presented a report upon the death of Homer Judd, of St. Louis, a member of the Association for years. The resolutions expressed regret at the loss to the profession and the Association of so valued a member, and contained a message of condolence to the bereaved family.

Dr. Truman requested permission to make some additions to the memorial of Dr. Homer Judd, as there were some facts connected with his life-work that he desired to procure and which were not at his command when he prepared it for the committee. The request was, on motion, complied with.

At the close of the session, Friday morning, the convention adjourned. A number of the members of the Association remained at the Elms until Monday, others made an excursion to the Rocky Mountains and the Pacific Coast, but the great majority returned to their homes and their offices well pleased with the Excelsior Springs convention.

